# The Impact on Parents of Diagnosing PCD in Young Children

**DOI:** 10.3390/jcm11164774

**Published:** 2022-08-16

**Authors:** Corine Driessens, Siobhan Carr, Edel Clough, Fiona Copeland, Sharon Dell, Lucy Dixon, Amanda Harris, Rebecca Knibb, Margaret Leigh, Manjith Narayanan, Beatrice Redfern, Evie Robson, Michael Sawras, Lynne Schofield, Kelli Sullivan, Myra Tipping, Nhu Tran, Woolf Walker, Jane S. Lucas, Laura Behan

**Affiliations:** 1 University of Southampton Faculty of Medicine, School of Clinical and Experimental Sciences, Southampton SO17 1BJ, UK; 2 NIHR Applied Research Collaboration Wessex, University of Southampton, Southampton SO17 1BJ, UK; 3 Primary Ciliary Dyskinesia Centre, University Hospital Southampton NHS Foundation Trust, Southampton SO16 6YD, UK; 4 Primary Ciliary Dyskinesia Centre, Royal Brompton & Harefield Hospital NHS Foundation Trust, London SW3 6NP, UK; 5 PCD Support UK, Buckingham MK18 9DX, UK; 6 Department of Paediatrics, University of British Columbia, Vancouver, BC V6H 3V4, Canada; 7 Department of Psychology, School of Life and Health Sciences, Aston University, Birmingham B4 7ET, UK; 8 Department of Paediatrics, University of North Carolina at Chapel Hill, Chapel Hill, NC 27599, USA; 9 Leicester National Primary Ciliary Dyskinesia Diagnosis and Management Service, University Hospitals of Leicester NHS Foundation Trust, Leicester LE1 5WW, UK; 10 Primary Ciliary Dyskinesia Centre, Leeds Children’s Hospital at Leeds Teaching Hospitals NHS Foundation Trust, Leeds LS1 3EX, UK; 11 Hospital for Sick Children (SICKKIDS), University of Toronto, Toronto, ON M5G 1X8, Canada; 12 Marsico Lung Institute, University of North Carolina at Chapel Hill, Chapel Hill, NC 27599, USA

**Keywords:** primary ciliary dyskinesia, diagnosis, caregiver burden, chronic illness, preschool, COVID-19, quality of life

## Abstract

Primary ciliary dyskinesia (PCD) is an incurable, rare, inherited, chronic condition. Treatment includes the regular clearing of airway mucus, aggressive treatment of infections and management of hearing loss. Caregiver burden has not been explored, hence we interviewed 18 mothers and 6 fathers of children under 6 years to understand the impact of diagnostic testing and implications of a positive diagnosis. Interviews were transcribed and thematically analysed and five key themes were identified. These included the parents’ experiences following child’s diagnosis, impact of child’s treatment regimen on parent, impact of child’s health status on parent, parent’s coping strategies, and parental concerns for the future. Parents described their diagnostic journey, with the findings revealing how a lack of awareness among clinicians of the PCD symptom pattern can lead to a delayed diagnosis. Parents discussed the emotional and practical impact of a PCD diagnosis and the coping strategies employed to deal with challenges arising following a diagnosis. Parents use a variety of different lifestyle changes to accommodate their child’s treatment regimen and to cope with disruptive life events such as the COVID-19 pandemic. This study provides valuable insights into parental adjustment and adaptation to a PCD diagnosis and management regimen. Going forward, this research highlights the need for integrated social care for PCD patients and their families.

## 1. Introduction

Primary ciliary dyskinesia is a rare (1:10,000) genetic lung disease caused by impaired clearance of mucus and debris from the airways. Individuals with PCD often present with unexplained respiratory symptoms, requiring respiratory support [1]. They continue to have persistent lung and nasal symptoms in infancy, with frequent infections. Progressive chest symptoms persist throughout life and include a daily wet cough and recurrent chest infections almost invariably leading to irreversible bronchiectasis by adulthood [2]. Cilia do not clear fluid from the middle ear causing frequent ear infections, hearing impairment, delayed speech and learning. Dysfunction of the embryonic nodal cilia causes situs inversus (mirror image positioning of organs in the body) in ≈50% of cases, sometimes associated with congenital heart disease [3].

Despite symptoms from birth, diagnosis is often delayed. This has been attributed to a number of factors including a lack of PCD awareness amongst clinicians and failure to take past history into account [4]. Additionally, diagnosing PCD is complex, requiring expensive infrastructure, and an experienced team of clinicians, scientists and microscopists. European guidelines recommend that PCD should be confirmed in a specialist centre using appropriate diagnostic testing [5,6]. To diagnose PCD, a combination of tests are used because a single test cannot reliably diagnose all PCD types. However diagnostic guidelines agree that abnormal ciliary ultrastructure assessed by transmission electron microscopy (TEM) and/or bi-allelic mutations in a known PCD gene is a definitive confirmation of a PCD diagnosis [6,7]. Major efforts to improve the knowledge of paediatricians has reduced the average age of diagnosis from 6 years in 2009 to 2.6 years in 2015 [8,9]. There is no cure, and management aims to control symptoms, improve hearing, treat infections and delay lung damage [10]. Most importantly, PCD patients need to adhere to daily time-consuming treatment airway clearance regimens aimed at facilitating mucus clearance [11]. There is currently no cure for PCD, and new treatments to correct dysfunctional ciliary movement due to mutations in ciliary genes are being studied [12,13].

From birth, the parents of children living with PCD are faced with the challenges of caring for a child with a (un)diagnosed chronic condition. Parents not only assist the child in activities of daily living, but also accommodate the nutritional, physical, social, emotional, medical and financial needs associated with the chronic health condition. This has been shown to differentially impact parental wellbeing. Pelentsov et al. (2016) for instance describes that parents of a child with a rare chronic condition felt socially isolated as others did not understand what the parent was going through [14]. Many mothers reported that caring for a child with a chronic condition required adjustment in their employment. Germeni et al. (2018) found that parents of children with chronic conditions try to reconstruct some form of normality once diagnosis has been made [15]. The stability of this normality depends heavily on the fluctuations in the health status of the child with the chronic health condition and the ability to control these fluctuations [16].

To date, the impact of child PCD on parental wellbeing has not been well documented. A previous study has indicated that a small group of Italian mothers of children (age 6 to 16 years) living with PCD experienced more distress and stress than mothers of children who did not have a chronic health problem; furthermore all PCD mothers were found to have stress scores at, or above, the 85th percentile [17]. Another study found that Turkish parents to children (age 6 to 16 years) diagnosed with PCD experienced significantly less caregiver burden than parents of children diagnosed with cystic fibrosis, despite having lower mean FEV1 scores. This study found no significant correlations between pulmonary function score and quality of life scores with caregiver burden among parents of children with PCD; the authors suggest this may demonstrate the lack of knowledge among caregivers about PCD, particularly on the importance of pulmonary function tests, and a lack of follow-up of PCD in this population [18]. A small number of previously conducted qualitative studies have shown agreement among PCD parents that barriers to completing treatments meant less freedom in life. They reported how other duties, such as caring for other children and their employment, could limit their ability to complete daily treatments [19,20,21]. As so little knowledge exists, we conducted this qualitative study to explore the impact of caring for a young child diagnosed with PCD, particularly focusing on the diagnosis process and impact of a positive result.

## 2. Materials and Methods

The study is reported according to the Consolidated criteria for Reporting Qualitative health research (COREQ) [22]. The study received ethical approval from the Southampton and South West Hants Research Ethics Committee (06/Q1702/10; University of Southampton ERGO#53155), University of North Carolina Internal Review Board (IRB#09-1099), and Hospital for Sick Toronto Children Research Ethics Board (REB#1000024263).

### 2.1. Recruitment

English-speaking parents of young children (<6 yrs old) who had received a diagnosis of PCD according to international guidelines [6], were recruited by a PCD specialist team from three PCD centres in England (Southampton, London, Leeds) and two PCD reference centres in North America (Chapel Hill, NC, USA; Toronto, Canada) between November 2019 and November 2020. Diagnosis in this sample was determined based on a combination of the following criteria: hallmark TEM defect, biallelic causative mutation in PCD genes, and hallmark HSVM alterations [6,23]. In addition, recruitment advertisements were circulated among the members of the PCD Support Group UK. Clinical teams recruited twenty-one parents, and three members of the PCD Support Group agreed to participate. This method of convenience sampling was used as PCD is a rare disorder; however, efforts were made to ensure a diverse range of ethnic backgrounds, socioeconomic statuses, and disease severity. If both parents agreed to participate in the study, they were interviewed separately, except in one case where the parents were interviewed together.

### 2.2. Participants

Eighteen mothers and six fathers of 20 children participated. Detailed information on the child is provided in Table 1. Most parents resided in the UK (*n* = 16), as well as 3 in Canada, 2 in USA, 1 in Georgia, 1 in Ireland, and 1 parent in the Netherlands. Four parents had completed secondary education, 3 completed further education, 6 completed professional training, and 11 completed higher education. Fourteen of the parents were part-time or fulltime employed, 2 of the parents were self-employed. Five of the parents self-identified as homemakers and three as unemployed.

### 2.3. Data Collection

Following consent, semi-structured interviews were predominately conducted with participants over phone, with a number also being conducted via videoconferencing. The interviewers found no substantial difference in the quality of either method. This is supported by a growing body of literature documenting telephone interviews as a viable and valuable mode for collecting qualitative data. Additionally they have been found to ensure geographical representation, provide a degree of anonymity and relieve pressures on participants to provide socially acceptable answers [24,25,26]. Interviews followed an interview guide exploring experiences around birth, child development and progression symptoms, healthcare needs of the child, impact of the child’s PCD on the parent, and thoughts about the future. CD, a psychologist experienced in mixed research methods, conducted most of the interviews. LB, experienced in developing Quality of Life scales for individuals living with PCD [17,18,19] and trained as a qualitative interviewer, conducted 3 interviews and validated thematic coding. The interview guide was developed by CD after a literature review and refined by a panel PCD clinicians and patient representatives (Box 1). Both interviewers were not known to any of the participants. Interviews with participants ceased when no new information emerged during the conversations. Data saturation was assessed periodically by CD and LB. All interviews were digitally recorded and transcribed verbatim. Participant’s identities were managed in a fully confidential manner: the participants were assigned a study number. Where parents mentioned their child’s name in the interview, this was replaced with ‘son/daughter’ in the transcript. Interview lengths ranged from 30 min to 125 min with most interviews lasting between 45 and 60 min.

### 2.4. Data Analysis

The transcripts were analysed using a computer software program: NVivo Version 12.0 (QSR International Pty Ltd., Melbourne, Australia). An inductive approach using reflexive thematic analysis was adopted [27]. The first 10 interviews were coded independently by CD and LB. Themes were identified inductively and similar subthemes were grouped together under an overarching thematic framework. The initial coding was cross-compared and a preliminary thematic framework was agreed upon. The remaining interviews were analysed by CD. The thematic framework was further refined by findings of the subsequent interviews and through discussion with the research team (Table 2).

Box 1 **Interview guide for semi-structured interviews.**

*General*

- Tell me about what is it like for you having a child with PCD?

*Diagnosis*

- When did you first recognise your child was experiencing symptoms caused by PCD?
- Can you tell me about how your child was diagnosed?
- What was your response when you learned your child’s diagnosis?

*Symptoms*

- Can you tell me about what symptoms your child experiences on a day to day basis?
- How do these symptoms affect your child on a daily basis?
- How does your child experiencing these symptoms affect you on a daily basis?

*Treatment burden*

- What type of things are you doing for your child’s PCD care?
- How do you incorporate your child’s treatment regimen in your daily life?
- When planning things like holidays or family activities are there things that have to be considered because of your child’s PCD?

*Care Received*

- Can you talk about your child medical care?
- Do you feel like you are being involved in the decisions that are being made about your son’s/daughter’s care?
- What words best describe what you feel like when your child is not feeling well

*Social Functioning and Role*

- Do you believe you have changed now that you are caring for a child with a chronic illness?
- How has having a child with PCD impacted on your relationships with others?
- Has having a child with PCD lead to new relationships in your life?
- How has having a child with PCD impacted on your personal, family or career goals?

*Emotional Functioning*

- What words best describe what you feel like now in the current situation?

*Concerns about the Future*

- Do you have any concerns about your child’s future?

## 3. Results

This paper concentrates on the impact of PCD on the parents whose child has received a positive diagnosis. Five themes created an understanding of the parent’s experiences following their child’s PCD diagnosis: impact of the child’s diagnosis on parent; impact of the child’s treatment regimen on parent; impact of the child’s health status on parent; parent’s coping strategies; parental concerns for the future. Under these overarching themes we identified several subthemes.

### 3.1. Impact of Child’s PCD Diagnosis on Parent

For those children with situs inversus, the abnormal organ arrangement was often picked up at the 20-week pregnancy scan. Parents described that once this was identified they were exposed to multiple prenatal tests and had to have regular appointments, with PCD being mentioned as a possibility during pregnancy or shortly after. Of the ten children with a situs abnormality, parents of 7 children (35%) credited the identification of abnormal situs to an earlier diagnosis for their child but also spoke of this as being a very difficult time (Table 3: Quote ID 1 and 2).

While all parents noticed the child’s symptoms at birth, only 11 children (55%) were diagnosed at this time. During the pre-diagnostic period, parents had no knowledge of how to support and care for their child and when reflecting on this, parents recollected their helplessness and emotional distress (Table 2 Quote ID 3). A number of parents (*n* = 5, 21%) reported frustration and feeling dismissed when raising the issue of their child’s symptoms to medical experts or to their extended family and friends. Parents discussed how medical experts lacked knowledge about PCD, with many parents being told their child would probably grow out of their symptoms (Table 3: Quote ID 4). These experiences differed from parents who already had experience of PCD. One mother who already had an older child diagnosed with PCD described the pre-diagnostic period of her older child as “terrifying” and “horrific” compared of her youngest child which was expected, although “sad” (Table 3: Quote ID 5).

Once the child was diagnosed, most parents felt mixed emotions. Feelings of relief came with having a name for their child’s condition, however, these mixed with feelings of sadness due to PCD being a life-long condition which would always need to be managed. Additionally, many spoke of feeling stressed (*n =* 5, 21%), overwhelmed (*n* = 6, 25%) and worried about their child’s future (*n* = 5, 21%) when they received the diagnosis (Table 3: Quote ID 6 and 7). For many parents (*n* = 7, 29%), they attributed feeling mixed emotions about their child’s PCD diagnosis, including relief on knowing what the condition is, and that the condition isn’t more severe. Parents spoke of meeting other families when their child was in hospital and feeling grateful that their child’s condition was manageable (Table 3: Quote ID 8). Some parents did not process their child’s PCD diagnosis immediately, but just focused on dealing with daily challenges. These parents described getting overwhelmed when faced with a major challenge, for example being told their child needs hearing aids (Table 3: Quote ID 9).

Parents spoke of the benefits of an early PCD diagnosis, primarily in how it has enabled them to begin treatment early and manage their child’s condition appropriately. In most cases it has allowed their child to access management services and receive the care they need. Relief was expressed once they had a label for their child’s symptoms and could communicate this to teachers, carers and relatives, all of whom were more aware and considerate of the child’s symptoms (Table 3: Quote ID 10 and 11).

### 3.2. Impact of Child’s Treatment Regimen on Parent after Diagnosis

Parents caring for their first child with PCD described feelings of worry and stress in having responsibility for PCD management. Treatment regiments were described as overwhelming, particularly in the early stages of PCD diagnosis, but became easier over time (Table 4: Quote ID 12). On diagnosis, parents changed their daily schedule to accommodate the child’s daily PCD treatment regimen with most parents providing their child with one to three daily airway clearance (physiotherapy treatment) sessions totaling 20 to 360 min each day. Parents scheduled or planned each day to accommodate the daily treatment or they developed a new daily routine (Table 4: Quote ID 13). Parents described having to wake up earlier in the morning and return from work or family excursions earlier in the evening to support their child with the PCD treatment. Parents described it taking 1 to 2 years following diagnosis to settle into a lifestyle that accommodated the medical needs of their child. While some parents shared the daily treatment burden, for most couples it was the mother who took responsibility (Table 4: Quote ID 14).

Parents generally prioritised treatment over any other activity. At this young age, where the child needs parental supervision to adhere to the daily treatment, compliance and adherence was reported as being challenging for parents. Some parents used a role model (older sibling, parent) to convince their children to do daily treatment. Some parents explained to their child that the treatment will keep the germs out and will make them feel better. For most parents repeated reminders, chasing, struggles, and restraining were commonplace. Other parents reported not making a big deal about it but handling treatment as something which had to be completed (Table 4: Quote ID 15). Parents also cuddled with their child or used other methods to calm the child so the daily treatment would not upset them. Various parents bribed their child to do daily treatment, but most parents used electronic distraction to encourage adherence to daily treatment (Table 5: Quote ID 16).

A number of parents felt unsure whether they were doing the daily treatment correctly and 11 (46%) parents reporting finding it hard and emotional to enforce treatment especially when the child was upset (Table 4: Quote ID 17). The parents described their frustration at the continuity of the condition and treatments. They explained how important it is for their child to do the treatment every day in order to stay well but felt time pressured to complete the PCD treatment before they did any other activities. The also described not being able to follow their own interests or schedule alone time (Table 4: Quote ID 18).

Similar to those who had a delayed diagnosis due to lack of awareness of PCD among medical practitioners, a number of parents (*n* = 9, 38%) also spoke of this challenge following diagnosis. Children with PCD are often prescribed antibiotics to treat repeated ear and lung infections. Parents discussed the difficulties they experienced in getting antibiotic prescriptions from a GP or having them filled by the pharmacy (Table 4: Quote ID 19). A few parents reported feeling worried about the side effects of antibiotics on their child’s health and they feared that antibiotics would stop working in the long run as their child was taking them quite often. One parent also discussed facing issues where their child became upset at having to take antibiotics due to the ‘nasty taste’ (Table 4: Quote ID 20).

In some healthcare settings children diagnosed with PCD attended a one-stop multidisciplinary clinic once every 3 months for review by a PCD respiratory consultant, respiratory physiotherapist, PCD nurse specialist, and sometimes a psychologist, dietician, and/or Ear–Nose–Throat consultant. In countries with fragmented PCD care, the parent was responsible for coordinating all appointments and communication with medical specialists needed for their child’s wellbeing. Most parents valued an update on the medical status of their child and felt involved in the medical decision making regarding their child. Parents felt they could contact their medical team whenever they had questions or issues, with one parent describing their medical team as family (Table 4: Quote ID 21).

In some instances, however, parents did not feel supported by the PCD healthcare team. A number of parents (*n* = 5, 21%) spoke of experiencing language barriers, unclear information, a lack of practical support, felt not listened to, or the parent felt powerless to refuse required PCD healthcare. Parents reported that once their child received a diagnosis, appointments were time consuming, sometimes requiring the parent and child to be at the hospital for several hours. Working parents often needed to claim a holiday or took unpaid leave to accompany their child to these appointments. For a few parents it was quite emotional to attend clinic (Table 4: Quote ID 22 and 23).

It could also be quite stressful due to the emotional/behavioural response of the child. Parents reported needing to prepare their child for the visit and often rewarded them for compliant behavior (Table 4: Quote ID 24). Parents reported trying to stay calm when they saw their children struggle with the procedures and did not like to restrain their child (Table 4: Quote ID 25). The worst time for parents was when their child was hospitalised. They described it as a draining time, full of emotions and anxiety where they felt out of control. Not being able to be together as a family and care for any additional children had an emotional impact on the parent (Table 4: Quote ID 26).

### 3.3. Impact of Child’s Health Status on Parent

In general, parents did not anticipate their child being born with health problems when they decided to start a family. Now that they were living with a child with a long-term health condition, they sometimes wished the child did not have a PCD diagnosis, were wondering why this happened to them or wished that there was a cure. Emotionally, the worries about PCD never left some parents. For some parents PCD worries were in the back of their mind, surfacing as soon as the child was feeling unwell, the child needed to visit a doctor or therapist, or when they received PCD-related correspondence. Parents wanted their child to live as normal a life as possible (Table 5: Quote ID 27)

Most couples had to adjust work commitments once their child was diagnosed with PCD. For eight of the parents interviewed, they or their partner stopped employment, started working from home, became self-employed, minimalised work hours, and/or took a less demanding vocational role. The other parent was no longer able to make overtime and needed to take holidays or unpaid leave to accompany the child to medical appointments. Losing this additional income financially affected most families living with a young child diagnosed with PCD. Parents felt that one of them needed to have the flexibility to attend to the child’s daily and emergency health needs. One mother explained that she started working part-time on account of getting run-down, tired, and needing to take too much time off (Table 5: Quote ID 28). Another mother explained that she had to stop working out of fear her children would pick up illnesses in other people’s care and how this had a major impact on her and her own life expectations (Table 5: Quote ID 29).

The child’s genetic diagnosis prompted three (13%) of the parents to reflect on family goals and some parents discussed their decision not to have any more children. One parent reported not having a second child as they did not want to risk passing the condition on to another child (Table 5: Quote ID 30).

For many parents, the PCD worries were with them every day. Always being on high alert was reported as being overwhelming. These parents felt worn out and overrun by PCD (Table 5: Quote ID 31). This distress had led to sleep deprivation, stress, and feelings of unhappiness, sadness, depression, and anxiety. Five of ten parents reporting mental health symptoms received a clinical diagnosis and/or treatment for depression and anxiety. One mother also reported how her physical health was affected and that she now received treatment for stress-induced high blood pressure.

On the practical side the parents did anything they believed would keep their child well such as being vigilant not to expose the child to infections, behaving more cautiously around other people, assessing the child’s health condition during the day as well as night, assessing the risk level of each activity, ensuring the child attended medical appointments, making sure the child had tissues to wipe their running nose, and prolonged breastfeeding. Most parents (*n* = 20, 83%) discussed being on constant alert monitoring their child and working to ensure they did not develop a cold or became unwell. A few parents checked their child with a stethoscope or oximeter on a daily basis, and many regularly measured the child’s temperature. Other parents kept a diary/record, or closely monitored the child’s symptoms. Parents mentioned a variety of emotions when child was unwell including that they were worried, scared, anxious, upset, or sad. One mother described her child becoming unwell very quickly and described hospitalisations as being horrific and overwhelming, and losing a sense of control (Table 5: Quote ID 32 and 33). 

Socially many parents felt their child’s PCD care did not leave any room for spontaneity or time for themselves. They did not get a break and were unable to go out much. PCD care interfered with their ability to see friends/colleagues/family and PCD limitations had changed family activities. They no longer had time for hobbies or even time to spend on their appearance. Although the PCD diagnosis of the child had placed a strain on many marriages, most couples had been able to work through this and came out the stronger for it (Table 5: Quote ID 34). In families with other children without PCD, parents sometimes believed these children lacked parental attention (Table 5: Quote ID: 35). Parents received support from friends and family, however, they reported feeling that nobody understood exactly what they are going through (Table 5: Quote ID: 36). 

### 3.4. Parent’s Coping Strategies following Diagnosis

There was wide variation in the way parents coped with the challenges of caring for a child with PCD. We have classified the different styles of coping into support seeking, problem-focused, emotional-focused, and cognitive-adaptive coping strategies. Some parents discussed how support from their partner, family, healthcare providers, and online PCD support groups had enabled them to cope when child was diagnosed, hospitalised or unwell. Some parents sought medical information to explain why their child was unwell while other parents needed to talk to somebody who was willing to just listen.

Several parents dealt with the challenges of raising a child with PCD pragmatically, by taking things day-by-day, and focusing on the problems at hand. They became knowledgeable about PCD and sometimes learnt new skills to take control (Table 6: Quote ID 37). 

Some parents tried to forget their worries and suppressed negative emotions that surfaced due to the challenges of raising a child diagnosed with PCD, for example by crying, exercising, keeping busy, thinking positive thoughts, having fun and laughing a lot. 

Some found themselves taking prescribed mental health medication, whereas other parents positively re-assessed the situation so it no longer induced negative emotional responses through techniques such as praying, not thinking about PCD, wishing for the miracle of a cure, or knowing that today is a bad day but hoping that tomorrow might be a good day. The most popular form of adaptive cognitive coping was the parent comparing their situation with somebody who was worse off. This was reported by six (25%) parents (Table 6: Quote ID 38).

### 3.5. Parental Concerns for the Future

Following diagnosis, the most frequently raised concern for the future was fertility which was discussed by eight (33%) parents. Parents worried how fertility treatments would impact their child (Table 7: Quote ID 39) and whether grandchildren would inherit this genetic disorder. Parents who were thinking about the future mentioned their anticipation of explaining PCD to their child when they are old enough to understand (Table 7: Quote ID 40). The expected social impact of PCD symptoms and the child’s potential emotional response were also described (Table 7: Quote ID 41 and 42).

Another concern that was frequently mentioned (*n* = 11, 46%) was the progression of disease severity. Some parents worried that lung damage might lead to lung transplant, progression of hearing loss, and even PCD cutting their child’s life short. Currently, parents were in control of PCD treatment and adherence, but six parents discussed feeling worried about whether their child would continue to manage their treatments adequately when they are older (Table 7: Quote ID 43). Many parents (*n* = 10, 42%) thought about how PCD might limit the child’s social activities, development, and career goals (Table 7: Quote ID 44).

### 3.6. COVID-19 Pandemic and Caring for a Young Child with PCD

Three months into this project, the COVID-19 pandemic started. As we wanted to focus on everyday experiences of parents caring for a young child with PCD, our data collection stopped from March to July 2020, the first wave in the COVID-19 pandemic in the UK. COVID-19 was rarely reported among children diagnosed with PCD (Pederson et al., submitted) and as the world tried to return to some form of normality, the data collection for this study resumed from August to December 2020. The interviews did not focus on the effect of COVID-19 on caring for a child with PCD, but parents spontaneously brought up the impact of COVID-19.

Parents discussed how anxiety-inducing it is caring for a young child with PCD due to COVID-19. One mother described how she worried about her child going to school and that he wasn’t shielded anymore (Table 8: Quote ID 45). Some parents deferred the start of school for an additional year and all parents mentioned that they had deferred any holiday activities. Several parents mentioned that the COVID-19 pandemic had impacted their child’s social interaction outside of the immediate household, especially their ability to attend sport activities. COVID-19 caused an additional level of social isolation for the family caring for a vulnerable child. One mother discussed how shielding during the beginning of the COVID-19 pandemic impacted on the social development of her child (Table 8: Quote ID 46).

Most frequently mentioned was the enhanced vigilance around infections since the start of the pandemic, for example cleaning hands regularly, deterring the child from putting items into their mouths, washing clothes of family members who had attended outdoor activities, cleaning groceries coming into the house. General PCD care was sometimes delayed, and routine clinic appointments postponed or conducted online, which especially impacted children with hearing problems and speech delays (Table 8: Quote ID 47). 

## 4. Discussion

To the best of our knowledge, this is the first study exploring parental experiences of caring for pre-school children diagnosed with PCD. It provides valuable insights into parental adjustment and adaptation to the diagnosis and management of their child’s PCD. Diagnosis of this rare and persistent condition was a disruptive life event. Adjustment to a new normality was a gradual process, facilitated by getting a diagnosis and an explanation for the child’s symptoms. Similar studies exploring how a child’s long-term condition is integrated in parental functioning reported that adjustment is a continual process. While caring for the child, the parent will need to balance on-going changes in the child’s long-term condition, changes associated with the child’s developmental stages, and the ever-changing needs to the family [28]. These studies suggest that support to meet the main parental caregiver needs is important in this continual parental adjustment process [29]. 

Some families lived in countries where PCD health care was coordinated through a multidisciplinary team, whilst other families received fragmented care. These parents reported that the coordination of appointments with different specialties was time consuming. The risk of uncoordinated health care is a lower quality of care, higher costs of care, and increased hospitalisations [30].

As the COVID-19 pandemic started part way through the study, the information collected also covers PCD care during this unpredictable stressful life event. Like many parents caring for a child with a long-term condition, the uncertainty of the COVID-19 pandemic increased parental stress and worry [31,32]. However, as seen in a study among Italian parents caring for a child with PCD, these stress levels did not significantly differ from parents caring for a healthy child during this unprecedented uncertain time [33]. 

As there is no cure for PCD, the health status of the child is dependent on PCD management. Parents found the additional effort to keep their young child healthy and happy was not only physically but also emotionally taxing. Although parents recognised the benefits of a PCD treatment regimen, similar to the reports of parents with other chronic conditions [21], the commitment provided challenges to the fulfilment of their social, vocational, and parenting roles. Parents were not able to attend social activities to a level previously enjoyed. The parent had to be more cautious in their interaction with others and PCD caring instructions prevented participation in social activities.

Consistent with the experiences of parents caring for children with other lifetime chronic conditions [22] the parent’s vocational role and functioning was often affected. As reported by other studies [23] it was usually the mother who experienced changes in work status. In this study the parents often reported a need for more flexible, less demanding vocational roles. Families were often financially affected by combined reduction in working hours, as well as efforts to save for future healthcare procedures (e.g., fertility treatments).

Most parents felt a constant worry for their child’s wellbeing. About 50% of the parents reported recurrent feelings of anxiety and depression and 20% of the parents disclosed formal mental health diagnosis or treatment. Although it seems that a lot of parents responded emotionally to the caregiving responsibility, the assumed prevalence of mental health problems from this informal source of reporting is similar to the existing lifetime mental health prevalence in the general population [24]. More formal psychiatric epidemiological testing among the caregivers of individuals living with PCD would be appropriate to determine a more accurate prevalence of mental health problems among this population.

### 4.1. Limitations

Although we recruited an international, diverse sample of parents caring for young children diagnosed with PCD, this group of parents might not be representative of the population of parents caring for young PCD children, as 11 of the 24 participants were recruited from Southampton Children’s Hospital UK and only 5 were recruited from North America reference centres. This was a result of the COVID-19 pandemic affecting the ethically approved in-person recruitment procedure followed by the specialist centres. The length of period needed to obtain ethical approval for virtual recruitment or to resume in-person hospital consultations differed per centre. The sample has also a greater representation from mothers, and most participants have a white ethnic background even though the prevalence of PCD is higher in other ethnic groups [25]. In addition, due to the partially retrospective aspect of the project, we cannot exclude the possibility of recall bias when the parents commented on historic events.

### 4.2. Practical Implications

The findings of this study reveal that lack of awareness of PCD by healthcare providers sometimes limits disease management as it impedes GP care, emergency care, and pharmaceutical care. Due to the rarity of PCD, the symptom pattern is not always recognised early in life, thereby delaying diagnosis. Over the past decade, diagnostic awareness campaigns have been able to lower the age of diagnosis of this rare hereditary chronic condition in some countries. It would be prudent to extend these awareness campaigns and improve the knowledge of health care providers about PCD and other rare diseases.

The impact of PCD care on the parent can be overwhelming at times. It induces time constraints and requires not only physical but also emotional efforts. The mental health and resilience of caregivers is important to the quality of care provided. The parents of children living with PCD are often unable to get a regular break from their caring responsibilities. Caregiver burden might be eased and more personalised care provided if PCD health care was integrated with social care, coordinating health care services with support for tasks of daily living and engagement with local community [26]. Currently PCD services are geared towards providing appropriate services to cover the child’s PCD medical needs, but some parents reported a need for respite care; support with practical implementation of PCD management at home; caregiver appreciation; financial advice and support; awareness of community resources. A more holistic PCD care is responsive to meet not only the health care needs but also cover the social care needs of the family living with PCD.

## 5. Conclusions

This is the first international study examining the experiences of parents whose young child has been diagnosed with PCD. Findings show that caring for a young child diagnosed with PCD requires the parent to make practical life changes such as changes in their daily routines and lifestyle. PCD also impacts the parent’s financial status as well as emotional wellbeing which came with the need of implementing a treatment regimen and coping with challenging insecurities and widespread misconceptions. The findings from this study can be used to advise clinical practice in the diagnosis and holistic management of pediatric PCD care. 

## Figures and Tables

**Table 1:** Child characteristics.

	*n* = 20 (%)
Gender	
Male	14 (70%)
Female	6 (30%)
Ethnicity	
White	15 (75%)
Asian	3 (15%)
Other	2 (10%)
Ages	
1 year	2 (10%)
2 years	4 (20%)
3 years	3 (15%)
4 years	5 (25%)
5 years	6 (30%)
Birth order of child	
First	8 (40%)
Second	9 (45%)
Third	2 (10%)
Fifth	1 (5%)
Siblings with PCD	
None	7 (35%)
Younger	1 (5%)
Older	5 (25%)
Missing	7 (35%)
Sibling without PCD	
None	8 (40%)
Younger	0 (0%)
Older	8 (40%)
Missing	4 (20%)
Situs abnormalities	10 (50%)
Congenital cardiac disease	4 (20%)
Prophylactic antibiotics	7 (35%)
Respiratory infection(s) in past year	19 (95%)
Hospital Admissions in past year	6 (30%)

Primary ciliary dyskinesia (PCD).

**Table 2:** Thematic framework.

	Parent’s Reaction to:			Parental:	
Major Themes:	PCD Diagnosis Child	Treatment Regiment Child	Health Status Child	Coping	Concerns for Future
Sub-Themes:	Prenatal experiences	Daily treatment: Management Parent–child interaction Emotional impact on parents	Emotional	Support seeking	Explaining PCD
	Feelings before	Medical procedures and medication	Health	Problem-focused	Social/emotional impact PCD
	Feelings after	Outpatient appointments	Behavioural	Emotional-focused	Progression PCD
		Hospitalisation	Vocational	Cognitive adaptive	Impact PCD on development, social activities, career goals
			Family goals		Impact PCD on fertility
			Social		Ability child to manage PCD

Primary ciliary dyskinesia (PCD).

**Table 3:** Quotes from parents on the impact of a PCD diagnosis.

Quote ID	Quote	Parent
1	“So, that then triggered lots of things put in place for as soon as he was born. So we had like heart tests and things like that and obviously one of the things that they tested for, was PCD … So it was just really quick. Like we were kind of aware that it was something that he might have even before, I think, I don’t know if PCD was mentioned before he was born, but pretty soon afterwards.”	Mother 4
2	“In antenatal stage at my 20-week scan. … Half-way through the scan the lady who was doing the scan kind of went quiet and then said that she needs to get her colleague … and I just straight away knew there was something wrong … They got me an appointment the following day to see a consultant and then we discovered that his stomach is placed on the other side. So, through the antenatal stage I had lots of scans, I went to see the doctor, cardiologist to check his heart. Then I had a c section … it was just horrendous … they took him out, I had him with me just a few seconds and they take him to intensive care unit because they had to check him”	Mother 15
3	“Nobody ever really knew what it was, so I think that was the hardest for us, those four years of just what could it be? Could it be something you know life threatening? Who knows what it could be … before he was diagnosed, I was always worried and sometimes I would put it in the back of my mind for a few weeks and not think much of it until he got like a really bad cold … we were always worried because we knew something was wrong.”	Mother 10
4	“And every doctor we went to was like “he will grow out of it” or “it is probably reflux.” He was on reflux medication, didn’t help. They always had some sort of excuse, they used to just shrug it off.”	Mother 10
5	“When our first born was diagnosed … it was horrific. I was petrified, I was scared, I didn’t know what was going on, we didn’t have a clue what we were facing, didn’t have a clue what they were testing her for, so it was very much a case of we were scared. Whereas with our current child we knew exactly what they were testing him for, we were 99% sure that he had it (PCD), so actually it was more sadness that our thoughts, our worse fears kind of being confirmed I guess.”	Mother 3
5	“we were relieved and sad at the same time. Because PCD, it’s something he will have for the rest of his life, and something he will have to deal with for the rest of his life. You know, we were sad that he did have a condition that would affect him for the rest of his life … we were happy because now he is being treated, so now we can actually get things with his diagnosis so we are relieved that we have one.”	Mother 10
7	“it completely overwhelmed me and as I said I had a bit of a breakdown and I was on medication for at least 1 year … uhm … and I had psychotherapy as well at the time which helped.”	Mother 5
8	“that is just how we feel about it really uhm not ideal but it could be a lot worse and we just take everyday at a time”	Mother 4
9	“I think the hardest thing we ever found to deal with was the hearing aids … Which was just because we’d coped so far, like he was 18 months when he had hearing aids in and I think that, at that point we were like, actually it’s ok to not feel ok about this. Whereas up until that point we were like ok, yeah, we can do this, we can do this!”	Mother 4
10	“I think that’s because the diagnosis was so early that there was no lung damage, you know it’s not like we’ve waited 8 years for a diagnosis and haven’t done anything about it in the meantime, so he’s had physio since pretty much he was born.”	Mother 3
11	“So we were happy because now he is being treated, so now we can actually get things with his diagnosis so we are relieved that we have one. And you know we have something that we can tell his teachers … Because his teachers, you know before he had it, we always had to explain it is not a cold, we don’t know what is wrong with him but he doesn’t have a cold. And you know how people look at you like are you sure?”	Mother 9

**Table 4:** Quotes from parents on the impact of the PCD treatment regimen.

Quote ID	Quote	Parent
12	“When we started treatment yes, it was like a big whole day, I was either preparing or giving a treatment, so there was nothing I could do, no cleaning, no dinner, nothing. And it was like how, can I do this like forever and this was really hard. But later, I was little faster and now it is like everything is getting better.”	Mother 8
13	“Living with PCD it’s a lifestyle really, it’s something that we fit it all into the daily routine.”	Mother 1
14	“ … it has to be timed very well and done in a specific order … we can’t just do things spontaneously, you know like a family with children with, who don’t have the condition, you know if they want to change it up at the last minute, they can do that, whereas we couldn’t, if we went out for a certain period time for example, I couldn’t just say, yeah let’s just stay it’s fine we’ll just stay later, because it might be that I don’t have a certain medication on me”	Mother 3
15	“ … he does have this aerobika thing that he does after the nebuliser, so it’s like, it’s this whole process, he does it twice a day but he’s used to it, we don’t make a big fuss about it, … and then antibiotic, but he doesn’t know what it is, what it does. He just knows he has to take it.”	Father 4
16	“I mean we always put him in front of the telly and do and try and to make it like a nice thing, rather than something boring, so you know it’s part, his bedtime routine it works brilliantly because it’s like 10 min of chill out calm before stories and bed, so that works really well, but in the morning he just wants to play now, he doesn’t want to sit down or do it, so he does do it, but it’s bribed with you can have this at the end of it (Laughter) anything to get him to do it.”	Mother 4
17	“ … it is about emotionally as well, it wasn’t really comforting … she is looking at you and you are actually giving her physio and you want her to have the head mask as well and you know she doesn’t know what is happening … to be honest that was actually really really difficult. … she was getting very very edgy, like she was crying and really upset and we were supposed to keep her there for like 5–10 min, I mean, depending on how we feel, like if she is coughing or not.”)	Mother 2
18	“ … it’s harder for me in this aspect, to not to be able to go whenever you want … the thing for me is that I don’t have enough time to do everything what I want to do … I am more at home, at home and taking care of my kids, that’s what changed I guess. I‘m not going out as much as I used to”	Mother 8
19	“ … to get a new prescription, and if that’s something different I have to explain again, and then sometimes they need to call the specialist, … sometimes the doses is different than they used to give, it’s higher … sometimes the treatments are for four weeks instead of one and they’re like ’we need to ask questions’, I understand that, but sometimes I get so tired of explaining myself again.” (Mother 13)	
20	“ … one of the things which is quite difficult is to get to take his antibiotics. … as he’s got older that’s become more tricky and we’ve had to hide it in food. Because sometimes you’ve got to give him this stuff 3 times a day and it’s not particularly flavored nicely, so he’s very aware that this is medicine that he’s having and he doesn’t want to have it so, um obviously that’s something that is, you can overcome it but sometimes it’s quite tricky.”	Father 2
21	“They have been so awesome and I feel so close to all of them. They are almost like family now (laughs) … I can’t say anything negative about them. I feel included in every decision.”	Mother 13
22	“I find it quite difficult because it is a reminder I think of how serious his condition is and I struggle with going … it would just bring up a lot of emotions and I’d get really upset and I think I get really fearful even though they are a really … they’re really supportive … having a whole team of people was frightened me at first and … uhm … it … because it made everything so … made it even more serious...and more scary but...I knew that they were there for … for a good reason, I did not want my son to have all this … need, all these people”	Mother 7
23	“ … it affects mine and my partner’s life, so our jobs, because we have to take a lot of time out for hospital appointments or like every 3 months I have to have 2 weeks off at work.”	Mother 22
24	“ … sometimes when I say ‘hospital’ he thinks he’s being hospitalised, and that’s something in his mindset. If he says something like that, it makes me a bit sad. I have to explain to him which hospital it is, it’s really important to prepare him for that. Yeah, to prevent that he … he thinks he has to be hospitalised … I have to reward him afterwards. And we try to make it as positive as possible”	Mother 13
25	“The hard bits I suppose are when (child’s name) has to undergo medical bits and pieces like nasal brushings and things that he doesn’t want to do because either it hurts or it’s just an invasion of his privacy”	Father 2
26	“It’s horrible, it’s the worst time … I used to think it was hard when (child) ended up in hospital and at the time that was like the end of the world. You know, we had one child and that child was in hospital and you worried about that child and it was just awful. But actually, now having 2 with it and one without—having 3 children, what actually now has materialised is hideous, as a mother having to pick between your children, having to choose between them.”	Mother 3

**Table 5:** Quotes from parents on the impact of the child’s health status.

Quote ID	Quote	Parent
27	“I want my children to experience as normal a life as they possibly can and so for me, that includes travelling. So you know, yes we take those risks and we just go for it! And we do it because, you know I don’t want to have the regret of not having done it.”	Mother 3
28	“I work part time, I work 2 days a week, because after my daughter got diagnosed with PCD it was just too much I just couldn’t cope working any more than 2 days, um I just was really run down and tired and taking too much time off. I wasn’t sleeping well, I had to go down doing 2 days. Even so I do 2 days, I’m still struggling, I’m just thinking I might have to give up work, but financially we can’t cope to be honest if I did sort of give up work.”	Mother 14
29	“I stopped working so I could be here so they wouldn’t be at day care and wouldn’t get exposed to so many germs … you know I gave up my career so that I could better take care of the kids … I don’t work anymore. So this is the worst thing for me, if we are talking about me as a mother. I always thought that I would have a career and a family too, close together and right now I can only I don’t have any career right now and I studied a lot and when I meet my old friends, you know from university or from school, they are like, oh my god, I don’t believe you don’t work! This is what everybody expected or I was expecting from myself.”	Mother 8
30	“ … we always thought we have 2 children, but after having a child with PCD we decided not to have another child because we did not want to risk that we would pass the...the faulty genes on again and have another child with PCD...uhm I think very much...not so much from a selfish point of view as in we don’t think we’ll be able to cope with that … uhm … but we felt that it was not fair to risk having another child and passing on the condition again”	Mother 5
31	“I do feel I’m quite stressed, I’m quite run down. I just feel everything’s sort of on top of me at the moment … it’s just constant, sort of appointments and phone calls and ordering her medication and chasing it up and having to call this person and that person and so on and so saying they don’t know, then try this, it just goes round in circles sometimes, so I do get a bit frustrated and angry”	Mother 14
32	“With her it comes on so sudden, so you don’t really notice a change until it’s too late and you need to get her into the hospital on to oxygen. Because she could be fine, the last time we had it, she went to bed, she’d been at nursery all day, she had her tea, she went to bed, she was fine—I noticed she was a bit wheezy, so I gave her blue inhaler, I waited didn’t have any impact on her, gave it to her again and she was just getting worse and worse and worse. It was just so sudden like within a couple of hours we were having to call an ambulance out.”	Mother 11
33	“I hate it, it makes me cry every time. Because it just takes me back to when she was little when the first time we went into hospital and we were in there for 3 weeks. And it felt like we were never going to get out.”	Mother 11
34	“I think, for me and my husband, we’re a pretty good team actually. He, generally, in our general kind of marriage, we parent very similarly, but obviously it adds a bigger impact on us, on our marriage for sure because we’re making decisions that perhaps other parents don’t actually have to make. So it definitely puts a bigger impact on our marriage, but generally we’re pretty good at dealing with that.”	Mother 3
35	“I know it has had a big impact on my 2 other children, because of this condition that my child has PCD, you know. Because they can see I give her more time and I don’t have enough time for them and even if I do, I’m just too tired that my attention is not with them, especially when it comes to homework and just other stuff, you know cos I’m just physically run down and tired doing all the chest physio and stuff like that. I get tired myself, you know what I mean.”	Mother 14
36	“ … maybe they try to understand our position, but do they really get it? No, absolutely not! I don’t think, that’s a slight on anybody else, that’s just unless you’re living it, I guess you can’t really understand it. No more that I could understand somebody else’s position with perhaps a different condition.”	Mother 3

**Table 6:** Quotes from parents on their coping strategies following diagnosis.

Quote ID	Quote	Parent
37	“I’m actually qualified now, so I trained to do home IVs, so I can actually administer once they have a PICC line fitted and the consultant decides they’re ok to be in my care at home. I actually bring them home and I do the IV myself because again it’s given us the ability to back together as a family and I think that’s the biggest care—sometimes not being able to be together as a family.”	Mother 3
38	“I currently have in my team at work somebody who’s having a baby who’s got a tumor on her brain and it’s about viability of the baby, so when you think about what could be happening and that fact that my son has wonderful life and is very happy and he actually talks fine and he’s you know got a zest for life, he’s enthusiastic and he’s running around actually compared to perhaps what other people are going through, this really isn’t as big a deal as it could have been.”	Father 2

**Table 7:** Quotes from parents relating to concerns for the future.

Quote ID	Quote	Parent
39	“ … what I’m really concerned about also, is whether he’ll be able to have children one day. Because, I know the kind of science is moving in the right direction and there might be some further development, but from what I understand for males with PCD to actually conceive the child naturally it can be difficult. So he might need some extra help and I know that for some males that might be an issue, they won’t be an alpha male. Being able to fully, not just perform, but what I’m saying is being able to do all those things that they are kind of natural to male, and it might affect his confidence and self-esteem and mental health in the future.”	Mother 15
40	“I feel confident in terms that we’re doing the right thing at the right stage of his life at the moment. I feel nervous that we’ll get it wrong later on, especially as we have to start explaining things in more detail to him and I worry what his reaction with be.”	Father 2
41	“Currently my son is very confident, but kids can be very cruel and I think if he’s going to continue speaking very, very unclearly, they might pick up on him. … I don’t know, but you know simple things like, he can’t say finish, he’s saying pinish, and you know for 4 ½ its still quite cute, but when he’s 6, 8 or 10, it’s not going to be cute any more, it will become an issue for him”	Mother 15
42	“I think he will be more affected by it when other children get annoyed by it, so like he coughs a lot, you know and I can just imagine him sitting in class coughing and someone’s going “ooh, that’s not very nice”. So, it’s that, that we need to be like teaching him how to do it nicely, and make sure he blows his nose, and all of those things, just to support the social aspect of it. I guess it’s that side of it that needs to be carefully managed and supported as well because otherwise he will be physically fine but mentally not, so … we need him to be able to cope with that side of it.”	Mother 4
43	“ … my child will become more independent with treatments and stuff and we’re helping her to achieve that, but that’s quite tough to achieve that, because you want them to have the best possible treatment and in my eyes, the best possible treatment is me being in control of that treatment … So that concerns me. Have I brought her up in the best possible way I can for her to really comprehend how serious this condition is and that PCD if it’s well maintained … she can live … as normal a life as she allows herself to live, by the treatment she continues to do?”	Mother 3
44	“ … it’s going to have a big impact in her daily life, daily routine, because she will have to be home to do her therapy and stuff like that. She won’t be able to do all the normal things her friends do.”	Mother 14

**Table 8:** Quotes from parents relating to caring for their child during the COVID-19 pandemic.

Quote ID	Quote	Parent
45	“I think also, with COVID-19, that’s a completely different story. COVID-19 itself is really anxiety provoking and for someone who, you know their son has PCD, where we don’t fully know how he’s going to react because I understand this is pretty much case by case, the anxiety is even higher. He has to go to school because there isn’t a shielding group 3 anymore. God knows what he is going to bring back from that school … You know any kind of social contact, … outside of our house, so that’s even more frightening for us”	Mother 15
46	“ … when we had to keep him inside for like 3 months and I was at home with him, shielding that affected him and when we first came back to our lives and seeing other people he was really quite shy he didn’t want to see anyone again … that way of keeping him inside for so long it was hard for him to get back into society and stuff like with people again.”	Mother 17
47	“ … he had his hearing tested twice before lockdown before the March lockdown and then all the appointments got postponed. So, what they said is that he’s got a glue ear and he needs to be monitored closely but has not had any tests ever since”	Mother 15

## Data Availability

The data presented in this study are available on request from the corresponding author. The data are not publicly available due to the extent of data available.
